# Proceedings of the North American Society of Head and Neck Pathology Companion Meeting, New Orleans, LA, March 12, 2023: Classification of Salivary Gland Tumors: Remaining Controversial Issues?

**DOI:** 10.1007/s12105-023-01541-1

**Published:** 2023-05-15

**Authors:** Alena Skalova, Martin D. Hyrcza

**Affiliations:** 1 Department of Pathology, Faculty of Medicine in Pilsen, Charles University, Pilsen, Czech Republic; 2 Department of Pathology and Molecular Genetics, Bioptical Laboratory Ltd, Pilsen, Czech Republic; 3 Department of Pathology and Laboratory Medicine, Arnie Charboneau Cancer Institute, University of Calgary, Calgary, Canada; 4 Sikl’s Department of Pathology, Faculty of Medicine in Pilsen, Charles University, E. Benese 13, 30599 Pilsen, Czech Republic

**Keywords:** Salivary gland, World health organization classification, Intraductal carcinoma, Mucinous adenocarcinoma, Intraductal papillary mucinous neoplasms, Oncocytic carcinoma, Carcinosarcoma

## Abstract

The salivary gland section in the 5th edition of the World Health Organization Classification of Head and Neck Tumours includes a description of several new entities. In addition, numerous tumor variants were described and new concepts proposed, most of which have been based on recent molecular discoveries. However, there are still some controversial issues that remain to be resolved, and some of them are discussed in this review.

## Introduction

Salivary gland tumor pathology is one of the most challenging areas in all head and neck surgical pathology. The salivary gland section in the 5th edition of the World Health Organization Classification (WHO) of Head and Neck Tumours covers 15 benign and 21 malignant epithelial neoplasms, one benign mesenchymal tumor, and two non-neoplastic epithelial lesions, vast majority of which have been discussed and accepted unanimously during WHO Editorial Board Meeting [1]. There are, however, several problematic tumor entities, for which the consensual opinion was difficult to reach.

Intraductal carcinoma (IDC) of the salivary glands is rare and enigmatic tumor, the understanding of which is rapidly evolving particularly due to availability of molecular testing. Emerging molecular data suggest that IDC comprises at least three distinct subtypes, characterized by recurrent *NCOA4::RET* rearrangements in the majority of cases with intercalated duct differentiation, *TRIM27::RET* fusions in cases with hybrid features, and a complex genetic profile including *HRAS* and *PIK3CA* hotspot mutations in the apocrine subtype.

Although much has been learned about this IDC in the recent years, unanswered questions remain. Most controversial issue is the terminology and the biological potential of IDC. For example, the description of frankly invasive and metastasizing IDCs with canonical *NCOA4::RET* fusion casts doubt on its current name “intraductal carcinoma”.

Intraductal papillary mucinous neoplasms (IPMNs) were recently described as a distinct minor salivary gland tumor entity. Mucinous adenocarcinoma (MA) shows variable morphology and is defined as a primary salivary gland carcinoma that displays prominent intracellular and/or extracellular mucin. IPMN, however, shares many similarities with low-grade, papillary-cystic forms of MA, including the identical activating *AKT1* p.Glu17Lys (alias p.E17K) gene mutation, and therefore, it was originally proposed as a subtype within the spectrum of low-grade MA in the 5th edition of the WHO Classification of Head and Neck Tumours. Consensus has not been reached, and after a difficult discussion of the WHO Editorial Board, finally IPMN was not included. It is a matter of controversy if IPMN represents a low-grade form of MA, a non-invasive precursor neoplasm, or distinct entity.

There is no consensus about the existence of oncocytic carcinoma. Oncocytic appearance is a common change encountered in many different salivary gland tumors. Molecular studies have now shown many such tumors to be oncocytic variants of other salivary carcinomas. Therefore, for the 5th edition of the WHO classification, oncocytic adenocarcinoma does not have special category and has only been included in the emerging entity chapter.

In contrast, salivary carcinosarcoma has remained a separate entity despite controversial concept of the clonal relationship between highly heterogeneous carcinomatous and sarcomatous components.

## Intraductal Carcinoma with Invasion, is the 5th Edition WHO Name Again a Misnomer?

Intraductal carcinoma (IDC) is a salivary gland malignancy characterized by papillary, cribriform, and solid proliferations with predominantly intraductal but also invasive growth [1]. Previously termed “low-grade salivary duct carcinoma” [2, 3] and “low-grade cribriform cystadenocarcinoma” [4], IDC is recognized as a well-circumscribed unencapsulated neoplastic glandular and myoepithelial proliferations within ducts, with an expansive multilobular growth, resembling vaguely atypical ductal hyperplasia or low-grade ductal carcinoma in situ of the breast [5]. The cytology of IDC is predominantly bland but may show pleomorphism, composed of cells that may have cleared, eosinophilic, oncocytic, or apocrine appearance [5].

**Fig. 1:**
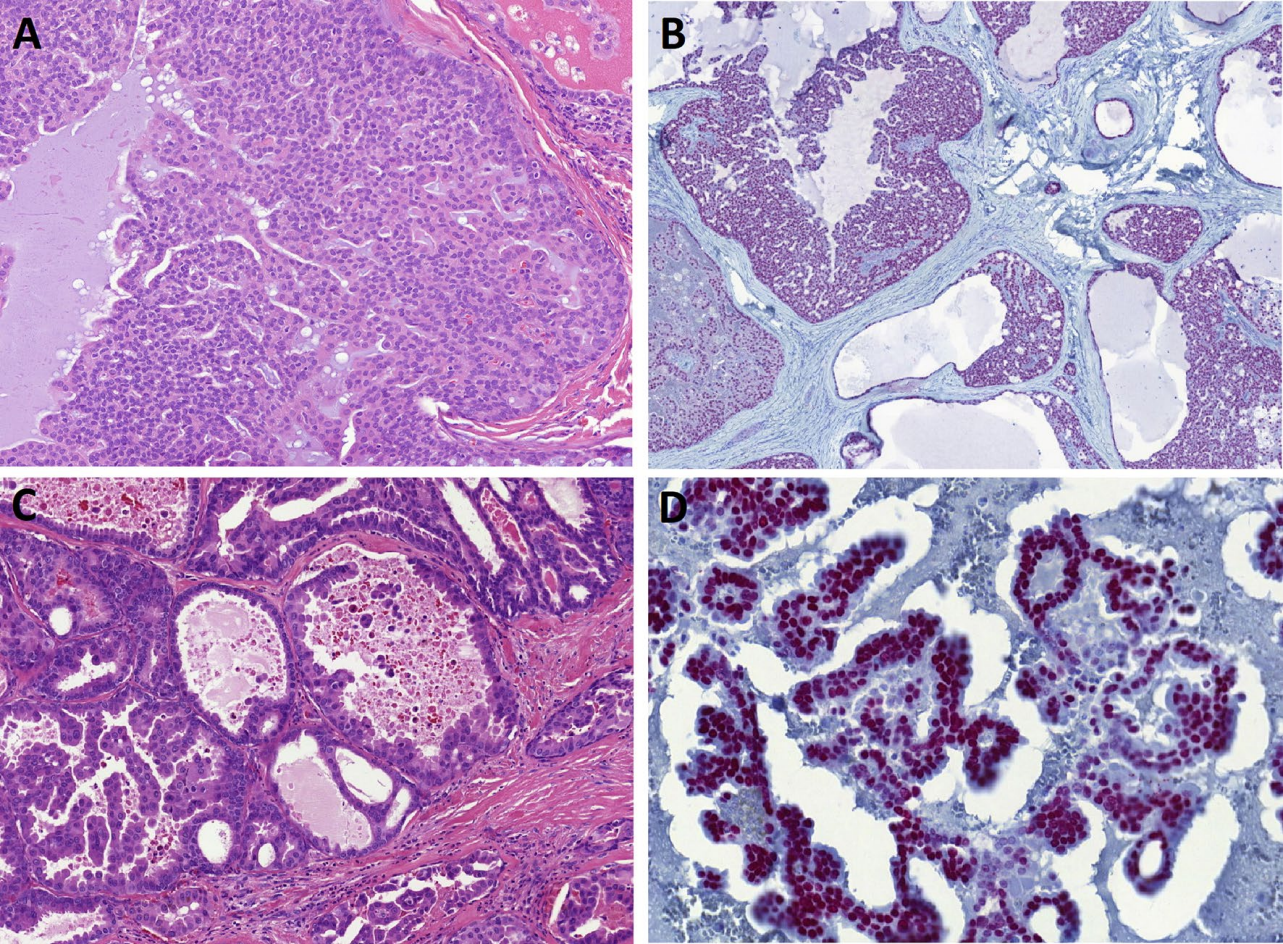
Intraductal carcinoma (IDC). Intercalated duct subtype IDC (**A**) which is SOX10 positive (**B**) and most commonly harbors *NCOA4*::*RET.* Apocrine subtype IDC (**C**) which is androgen receptor positive (**D**) and may harbor variable gene mutations in PI3K/Akt pathway

**Fig. 2:**
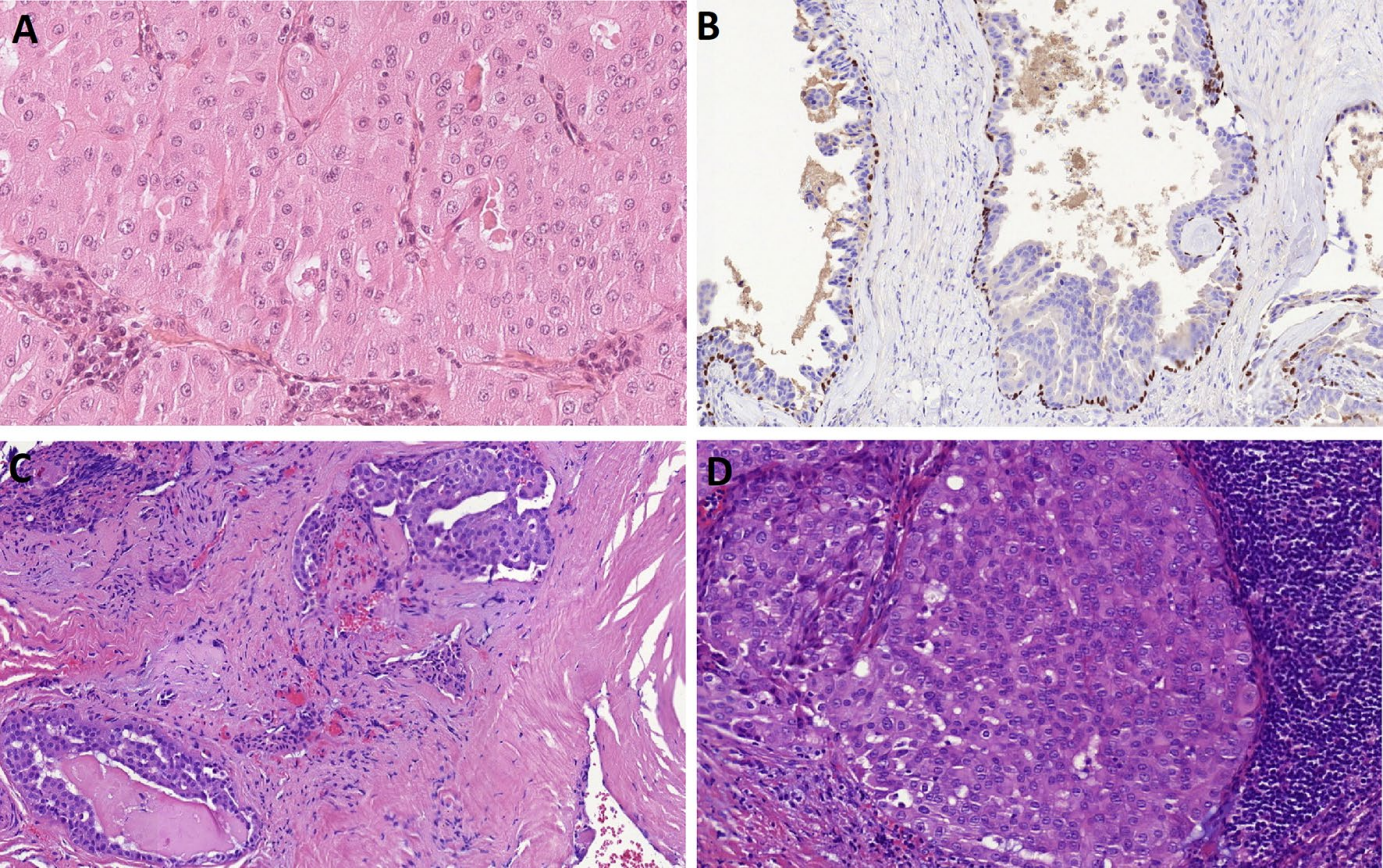
Intraductal carcinoma (IDC). Oncocytic IDC which resembles intercalated duct IDC but has more solid growth, prominent oncocytic cells, and more often it harbors a *TRIM33*::*RET* (**A**). In most cases of IDC a continuous layer of myoepithelial cells can be demonstrated by immunohistochemistry (p63 protein) (**B**). IDC with a loss of the myoepithelial layer and focus of microinvasion associated with desmoplastic reaction (**C**). Intercalated duct subtype IDC with *NCOA4::RET* fusion with widespread invasion and regional lymph node metastasis (**D**)

IDC is a morphologically heterogeneous tumor that contains at least three, maybe even four, usually distinct, despite sometimes overlapping entities. The most common pattern is an intercalated duct IDC which is S100 and SOX10 positive and most commonly harbors *NCOA4*::*RET* (Fig. 1A, B) [6, 7]; the second pattern is an apocrine IDC which is androgen receptor positive and may harbor variable gene mutations in PI3K/Akt pathway (Fig. 1C, D) [7–9]; less common pattern is an oncocytic IDC which resembles intercalated duct IDC but has more solid growth, prominent oncocytic cells, and more often, it harbors a *TRIM33*::*RET* (Fig. 2A) [10, 11]. Lastly, the mixed or hybrid IDC has components of more than one IDC subtype, and it often harbors a *TRIM27*::*RET* gene fusion [7, 12, 13]. It should be pointed out that all four IDC subtypes are currently only provisionally defined based on their histology and molecular changes, and their clinical significance is uncertain. It is possible that the rare oncocytic subtype may simply represent a collection of oncocytic variants of the other three IDC subtypes, rather than its own subtype. On the other hand, as outcome data for these tumors become available, some of the subtypes may eventually be established as separate entities.

Despite the differences in the luminal cells, all four types are currently included under the umbrella of “intraductal carcinoma” because the tumor islands are surrounded by a continuous layer of myoepithelial cell, which can be demonstrated by immunohistochemistry (Fig. 2B). By an analogy to breast ductal carcinoma in situ, the presence of this layer has been taken to mean that the carcinoma is still in the in situ phase, and by extension, when the IDC shows a loss of the myoepithelial layer with an associated desmoplastic reaction, this was interpreted as invasive carcinoma evolving from the intraductal carcinoma (Fig. 2C) [6, 13, 14] and the classification of “IDC with invasion” has become a major controversy [13, 14].

A recent study of frankly invasive carcinomas ex-IDC highlighted their remarkable diversity [14]. To complicate matters further, a single case of intercalated duct subtype IDC with *NCOA4::RET* fusion with widespread invasion and regional lymph node metastasis was recently published (Fig. 2D) [13], as were the cases of invasive IDC with intact myoepithelial cell layer invading the bone of the palate [15] and lymph nodes [16]. Moreover, another study demonstrated that the myoepithelial cells of IDCs harbor the same <em>RET</em> rearrangement as the more prominent ductal cells, which suggests IDCs may not be “in situ” neoplasms but rather biphasic salivary gland tumors with neoplastic ductal and myoepithelial cells [17]. For this reason, our group has recently proposed reclassifying IDC with intercalated duct phenotype as “intercalated duct carcinoma”, invasive or non-invasive [13]. However, this suggestion is not currently endorsed by the WHO, which recommends designating IDCs with invasive component as salivary adenocarcinoma, NOS, ex-IDC.

In summary, the taxonomy of IDC remains a matter of controversy, as reflected by the frequent terminology changes. The recent molecular characterization has demonstrated recurrent *RET* rearrangements, which are defining several different subtypes of IDC. Although apocrine variant of IDC is believed to be distinct from salivary duct carcinoma, recent developments have shown that they demonstrate similar molecular features in a subset of cases [6, 8]. For the time being, further studies are needed to decide if pure apocrine low-grade and high-grade IDC are related to salivary duct carcinoma, or if they merit a distinct diagnostic category [8].

## Intraductal Papillary Mucinous Neoplasm Versus Mucinous Adenocarcinoma, are they Cousins?

**Fig. 3:**
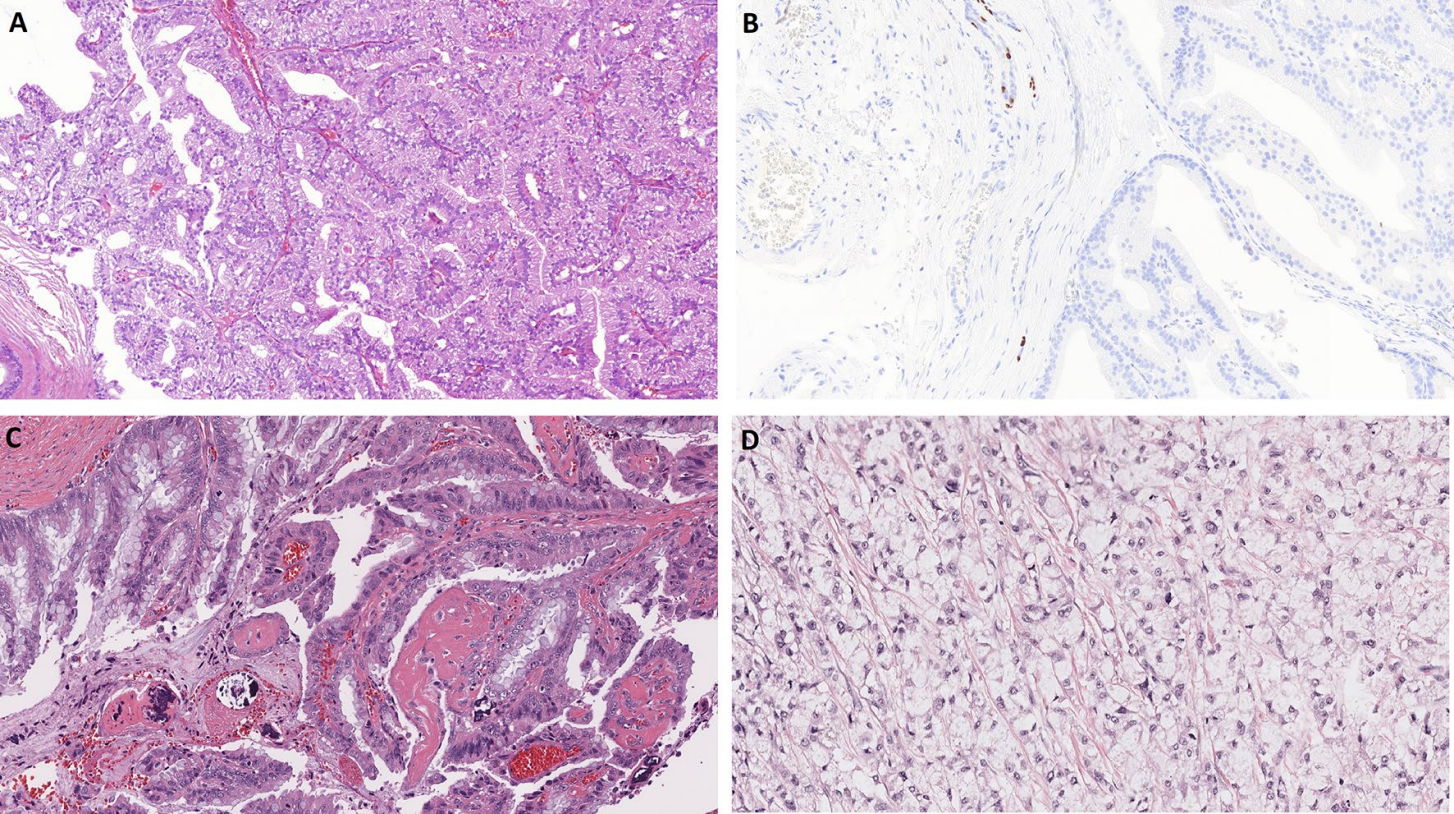
Intraductal papillary mucinous neoplasm (IPMN) and mucinous adenocarcinoma (MA). IPMN is a low-grade papillary-cystic tumor composed of mucinous columnar cells and harbors recurrent *AKT1* p.E17K mutation in most cases (**A**). IPMN lacks surrounding myoepithelial or basal cells (immunohistochemical staining, p63 protein) (**B**). Most MA is cystic and papillary, with pushing invasive borders (**C**). Occasional cases demonstrate signet ring or colloid (tumor cells floating in pools of mucin) patterns (**D**)

Low-grade papillary-cystic tumor composed of mucinous columnar cells was recently described as a distinct minor salivary gland tumor entity originally designated as intraductal papillary mucinous neoplasms (IPMN) [18, 19]. IPMN harbors recurrent *AKT1* p.E17K mutation in most cases [18] (Fig. 3A, B). Mucinous adenocarcinoma (MA) is defined as a primary salivary gland carcinoma that displays prominent intracellular and/or extracellular mucin [20]. MA is a histologically heterogeneous entity which include colloid carcinoma, papillary cystadenocarcinoma, and signet-ring cell carcinoma. Most MA are cystic and papillary, with pushing invasive borders (Fig. 3C). The lack of uniform histological appearance has prevented MA from its inclusion as a standalone entity in the 4th WHO edition of the classification; however, the discovery of a recurrent *AKT1* p.E17K mutation [21] changed this in the 5th WHO edition. Occasional cases demonstrate signet ring or colloid (tumor cells floating in pools of mucin) patterns, often in combinations (Fig. 3D). The clinical behavior seems to correlate with growth pattern, with colloid and signet-ring-rich cases showing metastatic capacity in contrast to the indolent papillary-cystic MA.

The terminology for salivary IPMN was proposed because the lesions were well-demarcated, mimicking intraductal proliferations [18]. However, most of the reported salivary IPMN cases lack surrounding myoepithelial or basal cells (Fig. 3B) suggesting that they are not true intraductal proliferations [20–24]. Furthermore, invasion into the surrounding parenchyma and lymphovascular invasion in salivary gland IPMN have been reported in several recent studies [24, 25]. Originally, IPMN was proposed as a subtype within the spectrum of low-grade MA; however, the members of the WHO Editorial Board could not reach a consensus on this issue and IPMN was, therefore, not included [20]. It is still controversial whether MA and salivary IPMN are separate entities or a disease spectrum, but because both MA and salivary IPMN are characterized by mucin production and the presence of activating *AKT1* p.E17K gene mutation, IPMN is currently considered by some to be a low-grade or non-invasive subtype of MA [22–24]. It is this author’s opinion that IPMN should be included within the MA spectrum, since the main distinction seems to be a lack of clear-cut invasion and a duct centric nature of IPMN. Whether these lesions should be regarded as indolent invasive papillary mucinous adenocarcinomas or as precursor lesions of an invasive mucinous adenocarcinoma remains to be established.

## Should Oncocytic Carcinoma Remain as Distinct Entity?

**Fig. 4:**
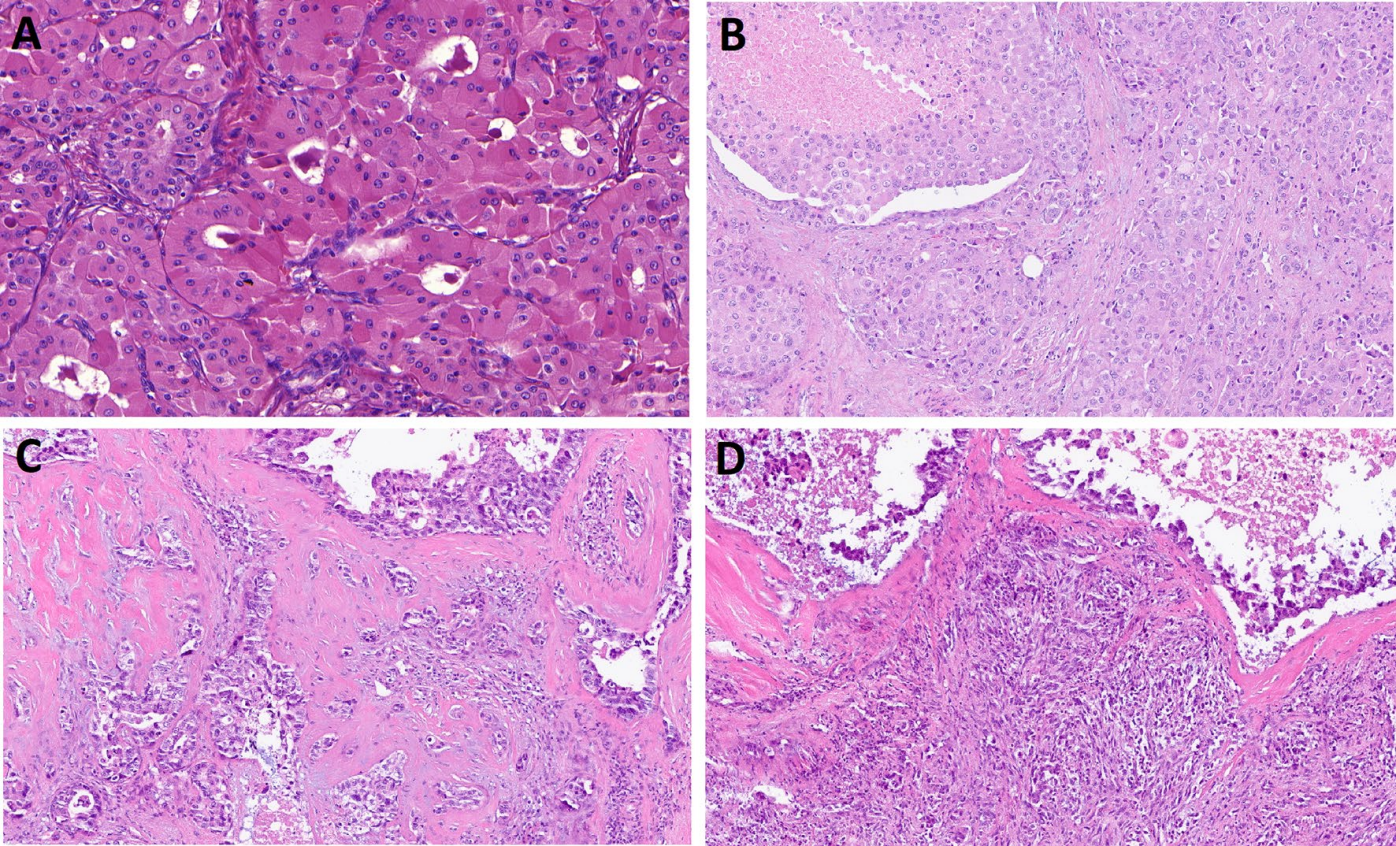
Oncocytic carcinoma and salivary carcinosarcoma (SCS). Oncocytic variant of mucoepidermoid carcinoma (**A**). Oncocytic variant of salivary duct carcinoma (**B**). SCS is characterized by variable combination of malignant epithelial and sarcomatous tumor components, which may develop from a pre-existent pleomorphic adenoma (PA) or de novo (**C**,** D**)

In the 4th edition, WHO Classification “oncocytic carcinoma” was defined is a malignant epithelial neoplasm composed exclusively of neoplastic oxyphilic cells which does not display any histopathological features of other specific salivary gland tumor types [26]. Nowadays, there is no consensus about the existence of oncocytic carcinoma. Oncocytic appearance is a common change encountered in many different salivary gland tumors. In the past, carcinomas consisting entirely of oncocytes were frequently diagnosed as oncocytic carcinoma. Molecular studies have now shown many such tumors to be oncocytic variants of other salivary carcinomas, such as oncocytic variant of mucoepidermoid carcinoma (Fig. 4A) [27], epithelial-myoepithelial carcinoma [28], and salivary duct carcinoma (Fig. 4B) [29], respectively. As such, it is unclear if oncocytic carcinoma exists as an independent entity and for this reason, the 5th edition of WHO classification has been moved from its own chapter to the emerging entity chapter [30]. This allows the pathologists a choice in the reporting of oncocytic carcinomas without detectable molecular alterations or ones in which the molecular analysis failed or could not be performed: those who believe oncocytic carcinoma does exist as its own entity can report it as oncocytic carcinoma, while those who do not, can report it as salivary carcinoma, NOS.

## Should Salivary Gland Carcinosarcoma Remain as Distinct Entity?

Salivary carcinosarcoma (SCS) is characterized by variable combination of malignant epithelial and sarcomatous tumor components, which may develop from a pre-existent pleomorphic adenoma (PA) or de novo [31]. It has not been clear whether the sarcomatous component of SCS represents a true sarcoma or an epithelial-to-mesenchymal transition in the carcinomatous component of a PA, and for this reason, the WHO board decided to retain SCS as a standalone entity in the 5th edition. However, a recent study by Ihler et. al. demonstrated that 15 of 16 carcinosarcomas in their series had evidence or their origin from a PA [32] (Fig. 4C, D). Based on this, it is this author’s opinion that SCS is a rare, but unique, and aggressive variant of carcinoma ex-PA with secondary sarcomatous overgrowth. In analogy to changes of terminology in other organs, the term “sarcomatoid carcinoma ex-PA with/without heterologous elements” might be more appropriate term in upcoming editions of WHO classification [32]. However, for this 5th edition, SCS has remained as a separate neoplastic category [31].

## Conclusion

Salivary pathology is a rapidly evolving field in which novel molecular discoveries have driven multiple changes in the tumor classification. While in most instances, the discovery of the molecular changes have helped clarify the classification, in certain instances, they had the opposite effect of obscuring the boundaries between entities and creating controversies. This is well illustrated in the case of IPMN versus MA, which both share the same *AKT1 p.E17K* mutation, which can be interpreted as supportive of merging these entities under one umbrella or as simply a case of two separate entities having the same mutation. Similarly, the molecular testing has allowed reclassification of most oncocytic carcinomas into other salivary carcinoma types leading to an unresolved question of whether there are any true oncocytic salivary carcinomas, and thus, if the entity should be retained as a standalone entity in the classification. Since the answer to this question is unknown, the entity has been placed in the emerging and provisional entities chapter for now. In case of IDC, the molecular discoveries suggest that it is a heterogenous group of tumors; however, whether they should all be considered subtypes of IDC or as separate entities remain to be seen. Finally, the recent molecular profiling of SCS suggests that the entity is likely a carcinoma ex-PA (CEPA) with sarcomatous transformation rather than a true sarcoma in most if not all cases, and this may lead to its reclassification as a variant of CEPA in the future editions of the WHO classification.

## Data Availability

The data that support the findings of this study are available from the corresponding author (AS) upon reasonable request.
